# Image to Fit the Clinical Picture: Point-of-care Ultrasound Assessment of Ebstein’s Anomaly in Peru

**DOI:** 10.5811/cpcem.2019.12.44783

**Published:** 2020-02-24

**Authors:** Ashley C. Rider, Andrea Dreyfuss, Roberto Inga

**Affiliations:** * Highland Hospital, Alameda Health System, Oakland, California; † Hospital Nacional Dos de Mayo, Department of Emergency Medicine, Lima, Peru

## Abstract

Ebstein’s anomaly is a congenital heart defect that when left untreated can lead to unique physical exam and ultrasound findings. This case describes a patient who presented with dyspnea and was found to have cyanosis, clubbing, and dilation of right-sided chambers on point-of-care ultrasound. The series of images highlights findings in late-stage Ebstein’s anomaly and serves as a springboard for the discussion of the pathophysiology, diagnosis, and treatment of this rare congenital heart disease.

## CASE PRESENTATION

**Image 1:**
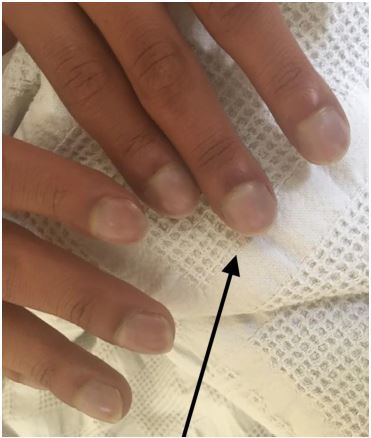
Fingers of patient (arrow) demonstrating cyanosis and clubbing.

**Image 2:**
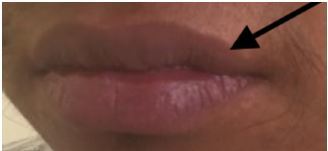
Mild cyanosis of lips (arrow) demonstrating chronic hypoxemia.

**Image 3:**
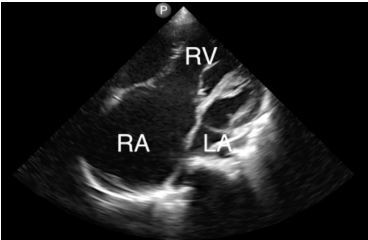
Point-of-care ultrasound apical 4-chamber view of patient with Ebstein’s anomaly demonstrating the dilated right heart chambers. The left ventricle is demonstrated by the arrow. *RV*, right ventricle; *RA*, right atrium; *LA*, left atrium.

A 20-year-old male presented to the emergency department with progressive dyspnea. He was noted to have hypoxemia, clubbing of the fingers (Image 1), and perioral cyanosis (Image 2). Point-of-care ultrasound revealed a severe anatomic abnormality of the heart consistent with Ebstein’s anomaly (Image 3 and video).

## DISCUSSION

Ebstein’s anomaly is caused by a congenital insufficiency of the tricuspid valve due to the apical displacement of the annulus. This leads to a dilated atrium and atrialization of the right ventricle as seen in this ultrasound image of a standard apical 4-chamber view.^1^ Other cardiac anomalies are commonly associated, such as atrial septal defect and ventricular septal defect.^2^

Ebstein’s anomaly accounts for less than 1% of congestive heart failure (CHF) and varies in severity.^3^ If tricuspid regurgitation is severe, symptoms such as CHF and cardiomegaly may develop in the neonatal period.^1^ Mild cases of Ebstein’s anomaly may remain undiagnosed until late childhood or adulthood, when presenting symptoms may include cyanosis and decreased exercise tolerance, as with this case. Adults also have a high risk of atrial tachyarrhythmia and ventricular pre-excitation, which predisposes patients to lethal arrhythmias.^4^

**CPC-EM Capsule**

What do we already know about this clinical entity?

Ebstein’s anomaly is a form of congenital heart disease caused by insufficiency of the tricuspid valve, leading to a dilated atrium and atrialization of the left ventricle.

What is the major impact of the image(s)?

These images show physical exam findings and point-of-care ultrasound (POCUS) features of late-stage Ebstein’s anomaly in a patient in Peru.

How might this improve emergency medicine practice?

In settings with limited access to pediatric cardiac surgery, patients may present with late manifestations of the disease. POCUS ultrasound may help in the diagnosis.

Patients with Ebstein’s anomaly may require medical or surgical treatment for atrialization of the right ventricle.^5^ Medical treatment includes diuresis, angiotensin-converting enzyme inhibitors, and digoxin. Tricuspid valve repair or replacement may be indicated in patients experiencing deteriorating exercise capacity, cyanosis (finger oxygen saturation <90%), paradoxical embolism, cardiomegaly, or reduction of right heart function.^5^ Surgical intervention should not be delayed until right heart failure occurs as this is associated with poor outcomes.^4^ Most cases of Ebstein’s anomaly fare well, especially when surgically corrected, with the majority of patients living to at least age 60.^3^

## Supplementary Information

Video An apical 4-chamber cardiac ultrasound obtained in a patient with Ebstein’s anomaly demonstrating the dilated right-sided chambers. The left ventricle is demonstrated by the arrow.
RV, right ventricle; RA, right atrium; LA, left atrium arrow.
